# Spatiotemporal Dynamics of Ecosystem Service Value Determined by Land-Use Changes in the Urbanization of Anhui Province, China

**DOI:** 10.3390/ijerph16245104

**Published:** 2019-12-13

**Authors:** Sai Hu, Longqian Chen, Long Li, Bingyi Wang, Lina Yuan, Liang Cheng, Ziqi Yu, Ting Zhang

**Affiliations:** 1 School of Environmental Science and Spatial Informatics, China University of Mining and Technology, Daxue Road 1, Xuzhou 221116, China; saihu@cumt.edu.cn (S.H.); long.li@cumt.edu.cn (L.L.); lnyuan@cumt.edu.cn (L.Y.); liang.cheng@cumt.edu.cn (L.C.); ziqi.yu@cumt.edu.cn (Z.Y.); tingzhang@cumt.edu.cn (T.Z.); 2 Engineering Research Center of Ministry of Education for Mine Ecological Restoration, China University of Mining and Technology, Daxue Road 1, Xuzhou 221116, China; 3 Department of Geography, Earth System Science, Vrije Universiteit Brussel, Pleinlaan 2, 1050 Brussels, Belgium; 4 School of Public Policy & Management, Anhui Jianzhu University, Ziyun Road 292, Hefei 230601, China; wangbingyi@foxmail.com; 5 College of Yingdong Agricultural Science and Engineering, Shaoguan University, Daxue Road 26, Shaoguan 512005, China

**Keywords:** ecosystem service value, land-use change, urbanization, spatio-temporal characteristics, Anhui province

## Abstract

Urbanization-induced land-use change will lead to variations in the demand and supply of ecosystem services, thus significantly affecting regional ecosystem services. The continuous degradation of ecosystem functions has become a serious problem for humanity to solve. Therefore, quantitative analysis of the corresponding impact of land-use change on ecosystem service value (ESV) is important to socio-economic development and ecological protection. The Anhui province in China has experienced rapid urbanization in recent years, and ecological environmental remediation and protection have become important goals for regional development. In this paper, the province of Anhui has been selected as a case of study, we analyzed the land-use change using Landsat images from 2000, 2005, 2010, and 2015. We then adjusted the equivalent factor of ESV per unit area and estimated the ESV of Anhui province from 2000 to 2015 to analyze the impact of land-use change on ESV. Our results show that (1) paddy field is the main land-use type in Anhui province, the built-up land area has continuously increased, and the water area has continuously decreased; (2) the total ESV of Anhui province decreased from 30,015.58 × 10^7^ CNY in 2000 to 29,683.74 × 10^7^ CNY in 2015 (the rate of change was −1.11%), and regulating services make the greatest contribution to ESV; and (3) land-use change has led to severe ESV variations, especially for the expansion of water area and built-up land. Our study results provide useful insights for the development of land-use management and environmental protection policies in Anhui province.

## 1. Introduction

Ecosystem services refer to the natural environmental conditions and functions of ecosystems and ecological processes that maintain human survival [1,2]. In addition, these are very important for regional and global ecological security, and are a frontier and focus of ecological and geography research [3]. Ecosystems support and sustain balance within the human living environment by regulating climatic conditions and maintaining biodiversity [4,5], providing food and raw materials for human life and reproduction, and providing entertainment and aesthetic enjoyment [2,6].

Land-use refers to the classification of space and time used by the main functions and activities that humans play on earth surface [7]. Land use change refers to the alterations (human-induced and not human-induced) that the land receives over the time [8,9]. Land-use has a considerable impact on the changes of ecosystem types, areas, and spatial distribution patterns, which are the most important driving forces of ecosystem service changes [10,11,12]. A loss of ecosystem services affects the stability of social development and human security and health, threatening regional and global ecological security [13,14]. Therefore, analyzing the correlation between land-use change and the spatial and temporal patterns of the ecosystem service value (ESV) can help predict the evolution trend [15]. This will improve the spatial structure of ecological land that aims to protect and stabilize regional ecosystems and can directly or indirectly perform ecological services [12] and the local ecological environment [16,17].

Numerous studies have previously evaluated the ESV [18]. Costanza et al. divided the global biosphere into 16 ecosystems and 17 ecosystem service types and then quantitatively assessed the global ESV [4]. This ESV assessment model is widely recognized and has been applied by many researchers. Furthermore, research on the impact of land-use change on ESV has increased significantly, including at the national-scale [19], regional-scale [20], basin-scale [21], and in multi-scale comparative studies [22]. Based on the research of Costanza et al. [4], Xie et al. conducted a questionnaire survey of 200 ecological experts [23] and then revised the ecosystem service types based on China’s ecological characteristics [24]. The revised ecosystem services include four primary types—provisioning services, regulating services, supporting services, and cultural services—and 11 secondary types—food production, raw materials production, water supply, gas regulation, climate regulation, hydrological regulation, environmental purification, soil formation and retention, maintain nutrient cycling, biodiversity protection, and recreation and culture. In addition, they developed and improved the equivalent factor of ESV per unit area in China, which has been used to evaluate the ESV in forest land [25], grass land [26], wet land [27], and areas of urban agglomeration [28]. This ESV assessment method is easy to use and provides highly comparable results. However, adequate consideration of the spatial and temporal differences in the ecosystem types and quality conditions is currently lacking. Therefore, the natural environment and socio-economic conditions of the study area should be fully considered in order to revise the equivalent factor of the ESV per unit area and to establish a suitable ESV assessment method for different ecosystem types and different ecological service types [29,30,31].

Since the beginning of the 21st century, the urbanization of Anhui province, China has accelerated, and the area of construction has rapidly expanded. From 2000 to 2015, the urbanization rate has increased from 28.00% to 50.50%. In addition, the growth of the population and economy in Anhui province has accelerated the development of the secondary industry and the tertiary industry and has promoted urbanization. This has led to changes in ecosystem structure and has threaten the stability of the ecological environment [32]. At present, a few ecologically fragile areas in Anhui province have been listed as extremely important regions for ecological protection by the Ministry of Ecology and Environment of the People’s Republic of China. Nevertheless, ecological environmental protection has become an important goal of regional development [33]. Therefore, a quantitative assessment of the impact of regional land-use change on the ESV is required for the sustainable development of socio-economic and ecological environment.

In this study, land-use change and spatiotemporal changes in the ESV were estimated quantitatively using Landsat image data and socio-economic statistics on Anhui province. The specific objectives of this study are as follows: (1) to analyze land-use change in the Anhui province in 2000, 2005, 2010, and 2015; (2) to characterize the spatiotemporal changes in ESV during the period 2000–2015 in different ecosystems; and (3) to explore the impact of land-use change on the ESV and provide a scientific basis for the formulation of land-use and ecological protection policies in the study area.

## 2. Materials and Methods

### 2.1. Study Area

**Figure 1:**
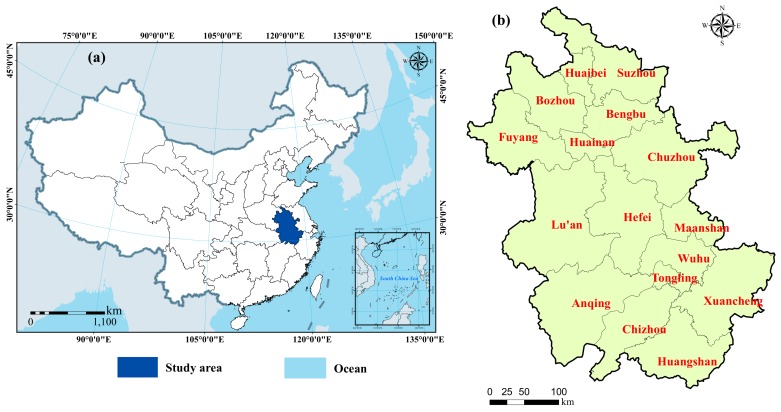
The study area: (**a**) the location of Anhui province in China; (**b**) the administrative division of Anhui province.

Anhui province is located in eastern China (114°54′–119°37′E, 29°41′–34°38′N), with a total area of 14.02 × 10^4^ km^2^ (Figure 1). Plains and low hills are the main types of terrain in Anhui province, which is characterized by a high altitude in the south and a low altitude in the north. The annual average rainfall in Anhui province is ~1300 mm, and rainfall is concentrated in summer and autumn, accounting for 40–60% of the total annual precipitation, which leads to frequent droughts and floods [33]. Anhui province is rich in water resources; the Chaohu Lake is one of the five largest freshwater lakes in China. The gross domestic product (GDP) of the region has increased from 290.21 billion CNY (Chinese yuan) in 2000 to 2200.56 billion CNY in 2015, according to the Statistical Yearbook of Anhui province for 2000, 2005, 2010, and 2015. As the economy has rapidly developed, urbanization in Anhui province has accelerated; the rate of urbanization increased by approximately 22.50% in the past 15 years. Rapid urbanization has led to dramatic changes in land-use, which has had a significant impact on the ecosystems of the region.

### 2.2. Data

The data that were used in this study include remote sensing images and socio-economic statistics. Considering the time interval and required image quality, multi-temporal remote sensing images from Landsat 5 Thematic Mapper (TM), Landsat 7 Enhanced Thematic Mapper Plus (ETM+), and Landsat 8 Operational Land Imager (OLI), acquired in 2000, 2005, 2010, and 2015 with a 30 m spatial resolution, were used in this study (Table 1) to monitor land-use change in the study area. All remote sensing data were downloaded from the United States Geological Survey (USGS) website. Prior to land-use classification, we performed data preprocessing, including radiometric calibration, atmospheric correction (FLAASH), geometric correction (image-to-image, mean position error ≤15 m, and no more than 0.5 pixels), and seamless mosaic, then we extracted the study area using the administrative boundary vector data.

The socio-economic statistics include population, GDP, grain producing area, grain production, and average grain price. These statistics were collected from the Statistical Yearbooks of Anhui province.

### 2.3. Methods

#### 2.3.1. Land-Use Classification

Maximum likelihood classification (MLC) method was used to classify the land-use type using the ENVI 5.1 (Exelis Visual Information Solutions company, New York, NY, USA) classic remote sensing image processing software package. According to the Current Land-Use Classification (GB/T21010-2017) [34] proposed by the Ministry of Natural Resources of the People’s Republic of China, the land-use types were classified into eight categories—paddy field, unirrigated field, forest land, grass land, water area, wet land, built-up land, and unused land.

Evaluating the accuracy of land-use classification is necessary in order to apply land-use changes [35]. High-resolution satellite images from Google Earth Pro were used to assess the accuracy of the land-use classification. Because of the lack of high-resolution images from 2000 on Google Earth Pro, we only verified the classification accuracy for 2005, 2010, and 2015. In addition, the remote sensing image data for the four periods were of the same type and were processed and classified using the same approach. Therefore, we infer that the classification accuracy for 2000 is similar to the classification accuracy for the other three periods. A total of 1000 sample points were randomly generated in the classified images in ArcGIS 10.1 (Environmental Systems Research Institute, Redlands, CA, USA) and then imported into Google Earth Pro to retrieve the ground truth data. By constructing a confusion matrix, the classification accuracy was calculated by programming in MATLAB [36,37]. The overall accuracies for 2005, 2010, and 2015 were 87.30%, 86.50%, and 86.90%, respectively; the kappa coefficients were 0.8529, 0.8355, and 0.8372, respectively. The kappa coefficients were all greater than 0.8, indicating that the classification results were acceptable and could be used for further analysis in this study.

#### 2.3.2. Quantitative Analysis of Land-Use Change

- Land-use dynamic degree

The land-use dynamic degree describes the change in the area of a land-use type during a specific period, which can reflect the speed of regional land-use changes, and plays an important role in the prediction of future trends in land-use change [38,39]. In this study, single and comprehensive land-use dynamic degrees were used to reveal the land-use change in Anhui province. The single land-use dynamic degree indicates the rate of variation for a specific land use type within a certain period in the study area, and the comprehensive land-use dynamic degree indicates the rate of variation for various land-use types within a certain period. The land-use dynamic degrees were calculated as follows:

(1) $$K=\frac{U_{b}-U_{a}}{U_{a}}\times \frac{1}{T}\times 100\%,$$

where K is the single land-use dynamic degree; $U_{b}$ and $U_{a}$ represent the area of a certain land-use type at the beginning and the end of a specific study period, respectively; and T represents the study period.

(2) $$LC=\left[\frac{\sum_{i=1}^{n}\Delta LU_{i-j}}{\sum_{i=1}^{n}LU_{i}}\right]\times \frac{1}{T}\times 100\%,$$

where LC is the comprehensive land-use dynamic degree;$\;\Delta LU_{i-j}$ is the absolute value of the converted area from the *i*th land-use type to the *j*th land-use type during the study period; and $LU_{i}$ is the area of the *i*th land-use type at the beginning of the study period.

- Land-use transition matrix

A transition matrix is a mathematical process based on Markov chain that is used in ecology; this process reveals information relating to the origin of the increase and the direction of transformation of the decrease in an area of a specific land use type [10]. The land-use transition matrix data was extracted by the dissolve, intersect, and calculate geometry tools in ArcGIS based on the land-use data and was calculated using the pivot table tool in Excel.

- Land-use intensity

The land-use intensity reflects the natural attributes of the land-use type, as well as the interference of human activities in the natural environment [40,41]. It is given by the following equation:

(3) $$L=100\times \overset{n}{\underset{i=1}{\sum }}A_{i}P_{i}/A_{T},$$

where L is the land-use intensity; $A_{i}$ is the area of the *i*th land-use type; $P_{i}$ is the classification index of the *i*th land-use type (of which unused land is one; forest land, grass land, wet land, and water area are two; paddy field and unirrigated field are three; and built-up land is four [42]); and $A_{T}$ is the total area of the land-use types.

#### 2.3.3. Evaluation of the ESV

In 1997, Costanza et al. [4] divided the global biosphere into 16 ecosystems and 17 ecosystem service types and then quantitatively assessed it. Based on the research of Costanza et al., Xie et al. (2015) adjusted and improved the value coefficient of China’s ecosystem services combined with China’s ecological characteristics [24]. The ESV has significant spatial heterogeneity over different regions due to the differences within regional ecosystems [29]. The table of the ESV equivalent factors proposed by Xie et al. is applicable at the national scale; however, if we apply it directly to regional ESV research, large errors may occur. Therefore, we adjusted the ESV coefficients to suit the ESV estimation (Table 2) and the ecological characteristics of Anhui province. The broadleaf forest and shrub grass land are the main types of forest land and grass land in Anhui province, we used the value coefficients of the former as the representatives of forest land and grass land, respectively [43]. Several researchers believe that built-up land does not belong to natural ecosystems and assign the ESV of zero to the construction land [18,19]. However, the basic functions of built-up land depend on ecosystems and have a negative impact on ecological service types such as water supply, gas regulation, and environmental purification [30]. In this study, we adjusted the value coefficient of built-up land based on the research by Kang et al. [31].

The equivalent factor of the ESV is determined by the relative contribution rate of the potential service value of the ecosystem, and its unit value is equal to one-seventh of the total economic value of grain yield per hectare of cultivated land [20]. Over the past 15 years, the annual average grain yield of Anhui province was 4727.1 kg/hm^2^, and the average grain price in 2015 was 2.33 yuan/kg. Therefore, the unit value of the equivalent factor of the ESV was 1573.45 yuan/hm^2^, obtained using the equation as follows:

(4) $$E_{a}=\frac{1}{7}\times P_{g}\times Q_{g},$$

where $E_{a}$ is the unit value of the equivalent factor of the ESV; $P_{g}$ is the annual average grain yield; and $Q_{g}$ is the average grain price.

Combining the value coefficients with the unit value of the equivalent factor of the ESV, we obtained the ESVs per unit area for Anhui province (Table 3). Then, the ESVs for different ecosystems were calculated using the following equations:

(5) $$ESV=\overset{n}{\underset{i=1}{\sum }}A_{i}\times VC_{i},$$

(6) $$ESV_{f}=\overset{n}{\underset{i=1}{\sum }}A_{i}\times VC_{fi},$$

where $A_{i}$ is the area of the *i*th land-use type; $VC_{i}$ is the ESV per unit area of the *i*th land-use type; $ESV_{f}$ is the value of the *f*th ecosystem service type; and $VC_{fi}$ is the ESV per unit area of the *f*th ecosystem service type of the *i*th land-use type.

## 3. Results

### 3.1. Land-Use Change Characteristics

#### 3.1.1. Change in Land-Use Structure

**Figure 2:**
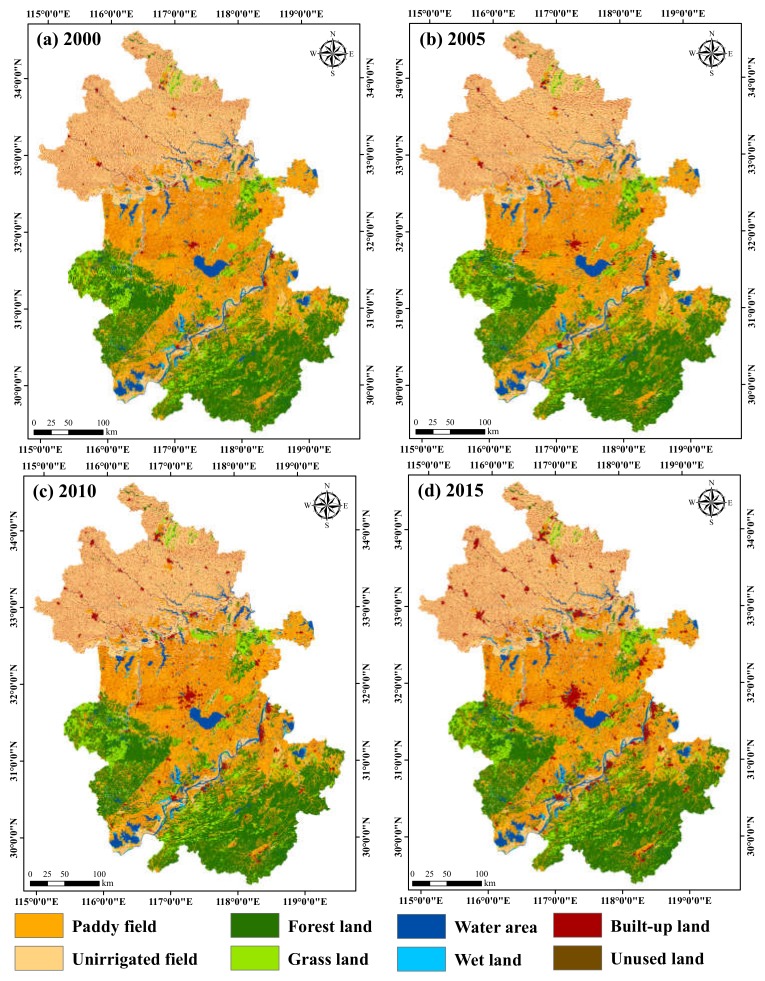
Land-use classification map of Anhui province.

Based on the interpretation of remote sensing images from the four periods, we obtained the spatial distribution and area variation (Figure 2) in land-use types from 2000 to 2015. We observed that paddy fields represent the largest proportions of land-use types, accounting for 31.26% (2000), 31.09% (2005), 30.43% (2010), and 29.60% (2015). During the period 2000–2015, areas represented by paddy field (from 43,812.94 km^2^ in 2000 to 41,483.58 km^2^ in 2015; a 5.32% decrease) and unirrigated field (from 36,866.44 km^2^ in 2000 to 35,443.04 km^2^ in 2015; a 3.86% decrease) have decreased significantly, while the area of built-up land has significantly increased (from 11,615.21 km^2^ in 2000 to 15,383.28 km^2^ in 2015, 32.44% increase). In addition, the water area slightly increased from 6095.34 km^2^ in 2000 to 6513.68 km^2^ in 2015 (a 6.86% increase), while there were little changes in other land-use types.

#### 3.1.2. Dynamic Degree of Land-Use Change

The single and comprehensive land-use dynamic degrees are shown in Table 4. The single land-use dynamic degrees show that paddy field, unirrigated field, forest land, and grass land areas continuously decreased. In addition, water area, built-up land area, and unused land area continuously increased. The land-use dynamic degrees of built-up land are greater than those of the other land-use types in the same periods, indicating that the built-up land area increased faster than other land-use types. Although unused land has a high single land-use dynamic degree, especially in 2010–2015, which reached 95.97%, its expansion is not significant, as determined by the original small area. The comprehensive land-use dynamic degree values significantly increased from 2000 to 2015, and the values in the periods 2005–2010 and 2010–2015 are 2.83 and 4.33 times that of the 2000–2005 period, respectively.

#### 3.1.3. Land-Use Transition

Table 5 shows the land-use transition matrix of Anhui province. The main land-use type transfer characteristics are as follows: Paddy field has the largest transfer-out area of 2848.69 km^2^, next are unirrigated field, which has a transfer-out area of 1654.34 km^2^; built-up land has the largest transfer-in area of 4048.75 km^2^, followed by water area, which has a transfer-in area of 594.70 km^2^. We found that the hot spot of transfer in Anhui province is the transfer of paddy field and unirrigated land to built-up land.

#### 3.1.4. Land-Use Intensity

We obtained the land-use intensity of Anhui province from 2000 to 2015 (Table 6) using Equation (3). The results show that both the land-use intensity and the growth rate continued to increase from 2000 to 2015. The land-use intensity increased slightly from 2000 to 2005, at a growth rate of 0.07%. However, over the next decade, the growth rate of land-use intensity significantly increased with growth rate of 0.4% in the period 2005–2010 and 0.5% in the period 2010–2015.

### 3.2. Evaluation of the ESV

#### 3.2.1. Changes in Total ESV

The ESVs of Anhui province in 2000, 2005, 2010, and 2015 were calculated in combination with the ESV per unit area (Table 7). The results show that the total ESV of Anhui province decreased from 30,015.58 × 10^7^ CNY to 29,683.74 × 10^7^ CNY, and the rate of change was −1.11%, during the 2000–2015 period. In addition, the total ESV for the 2000–2005 period increased to 30,015.71 × 10^7^ CNY and the rate of change was close to zero, while the total ESVs for the 2005–2010 and 2010–2015 periods decreased to 29,706.83 × 10^7^ CNY and 29,683.74 × 10^7^ CNY, with rates of change of −1.03% and −0.08%, respectively.

#### 3.2.2. Changes in the ESVs of Different Ecosystem Service Types

**Figure 3:**
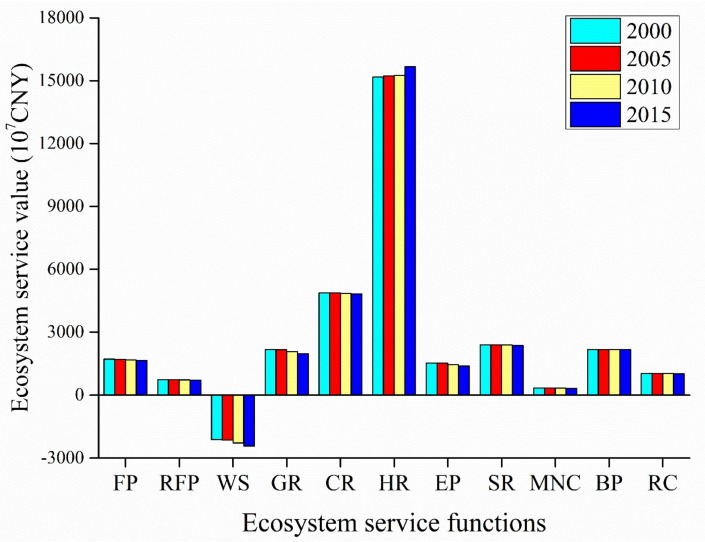
ESVs of different ecosystem service types in Anhui province.

Figure 3 shows the ESVs of different ecosystem service types. We sorted the four primary types of ecosystem according to their contribution to the ESV: Regulating service > supporting service > cultural service > provisioning service. Among the 11 secondary types, gas regulation, climate regulation, hydrological regulation, and soil formation and retention were the major ecosystem service types that affect the ESV. In particular, hydrological regulation made the largest contribution to the ESV and reached more than 50% of the total ESV in all four periods. In contrast, raw material production and maintaining nutrient cycling made the smallest contributions to the ESV (all below 2.5%). Moreover, water supply made a negative contribution to the ESV, and it continuously increased.

**Figure 4:**
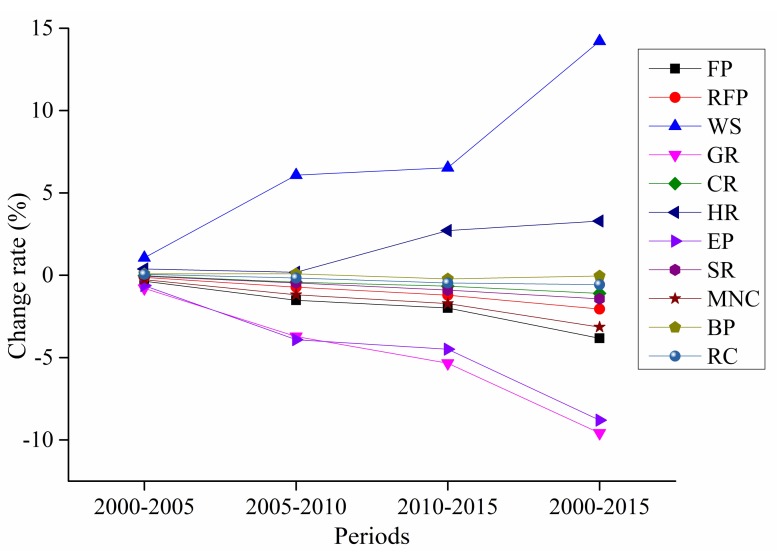
Contributions of different ecosystem service types to the ESV.

The dynamic changes in the contributions of ecosystem service functions to the ESV (Figure 4) show that food production, raw material production, gas regulation, climate regulation, environmental purification, soil formation and retention, and maintain nutrient cycling continuously decreased, while hydrological regulation and water supply continuously increased. The largest ESV rate of change was 14.21% for water supply, followed by gas regulation and environmental purification, the ESV rates of change for which were −9.58% and −8.80%, respectively.

#### 3.2.3. Effects of Land-Use Change on ESV

The largest ESV per unit area is for the water area and the lowest is for the unused land, whereas the ESV per unit area of the built-up land is negative (Table 3). We observed that the water area is the main contributor to the ESV of Anhui province and has contributed more than 40% during the four periods; the next highest contributor is the forest land, at approximately 39%. The contributions of the other land-use types, which have smaller areas, were relatively low, especially for unused land, which was close to zero. In addition, the ESV contributions of all land-use types show a decreasing trend, except for water area. Due to the small area of unused land, it was not considered here.

To clearly show the impacts of land-use changes on the ESV, we calculated the profit and loss matrix for the ESV (Table 8) using the land-use transition matrix (Table 5). The results show that the total ESV increased to 650.59 × 10^7^ CNY because of the land-use change, and the increase in water area contributed the largest added value to the ESV (1175.37 × 10^7^ CNY). Table 8 also shows that the built-up land that was converted from other land-use types led to a significant reduction (−765.10 × 10^7^ CNY) in the ESV from 2000 to 2015.

#### 3.2.4. Spatiotemporal Characteristics of ESV

In order to clarify the spatiotemporal characteristics of ESV in Anhui province, we calculated the ESV of all the 16 cities in Anhui province (Table 9). The proportions of the ESVs in the 16 cities were relatively stable in the four periods. We observed that the city with the largest proportion of ESV was Anqing, which has the largest water area, and all the proportions of the total ESV represented by the water areas are more than 23% over the four periods. In Lu’an, Xuancheng, and Huangshan, the proportions of ESVs are more than 10%. In addition, the cities with small proportions of ESV are Bozhou, Fuyang, Huaibei, Huainan, Tongling, and Suzhou, where the proportions are below 2% in the four periods.

**Figure 5:**
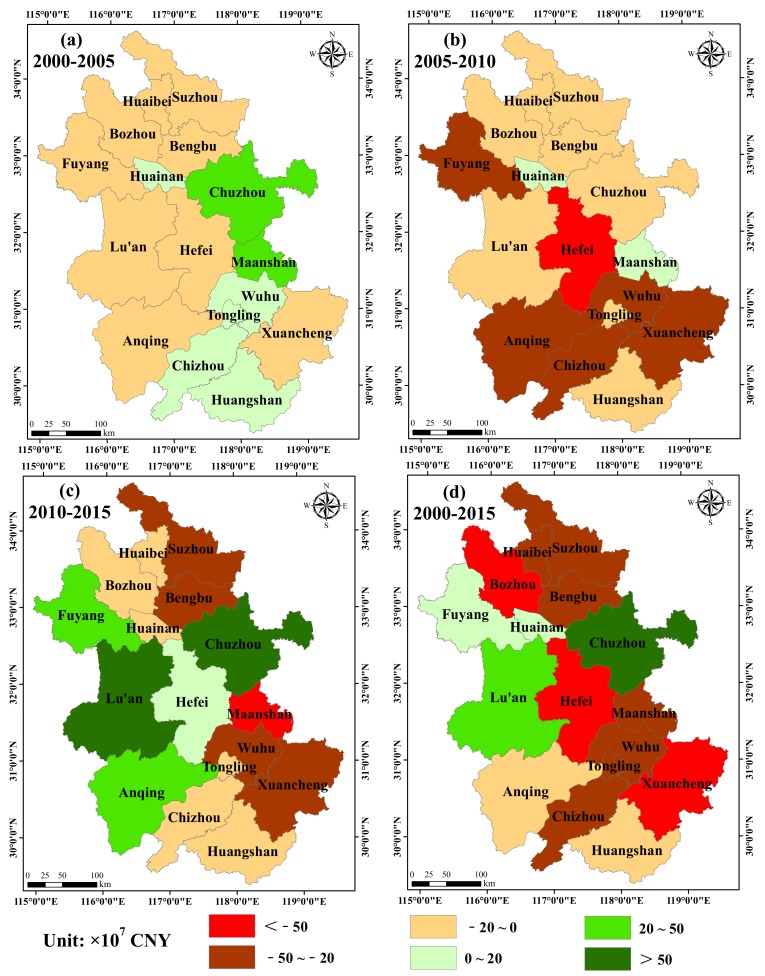
The changes in ESV in the 16 cities of Anhui province.

To show the differences in the ESVs in the 16 cities, the changes in the ESV were divided into six grades according to the increase and decrease in the ESV (Figure 5). In the first period (2000–2005, Figure 5a), there were six cities with an increased ESV and 10 cities with a decreased ESV, of which Maanshan showed the largest increase (35.01 × 10^7^ CNY) and Lu’an showed the largest decrease (−19.48 × 10^7^ CNY). In the second period (2005–2010, Figure 5b), the ESV of all cities showed a decreasing trend except for Huainan and Maanshan, and Hefei had the largest reduction −62.22 × 10^7^ CNY). In the third period (2010–2015, Figure 5c), five cities transitioned from decreasing ESV trends to increasing trends. In particular, the increase in the ESV for Chuzhou and Lu’an in the period 2010–2015 reached 66.64 × 10^7^ CNY and 55.79 × 10^7^ CNY, respectively, while Maanshan had the largest decrease of −69.09 × 10^7^ CNY. From the changes in ESV from 2000 to 2015 (Figure 5d), we observed that the ESVs of Fuyang, Huainan, Lu’an, and Chuzhou had increased, of which the largest increment was 75.21 × 10^7^ CNY in Chuzhou, followed by 27.97 × 10^7^ CNY in Lu’an. However, the ESVs of the other 12 cities were found to have decreased. In particular, Xuancheng and Hefei showed a significant decrease of −66.35 × 10^7^ CNY and −63.27 × 10^7^ CNY, respectively.

## 4. Discussion

In this study, we obtained land-use information for Anhui province for 2000, 2005, 2010, and 2015 using multi-temporal Landsat image data and characterized the dynamic changes in land use. In addition, we calculated the ESVs of these four periods and analyzed the response of the ESV to land-use change. The interpretation of the results and their implications are detailed below.

### 4.1. Spatial Distribution Pattern of ESV

**Figure 6:**
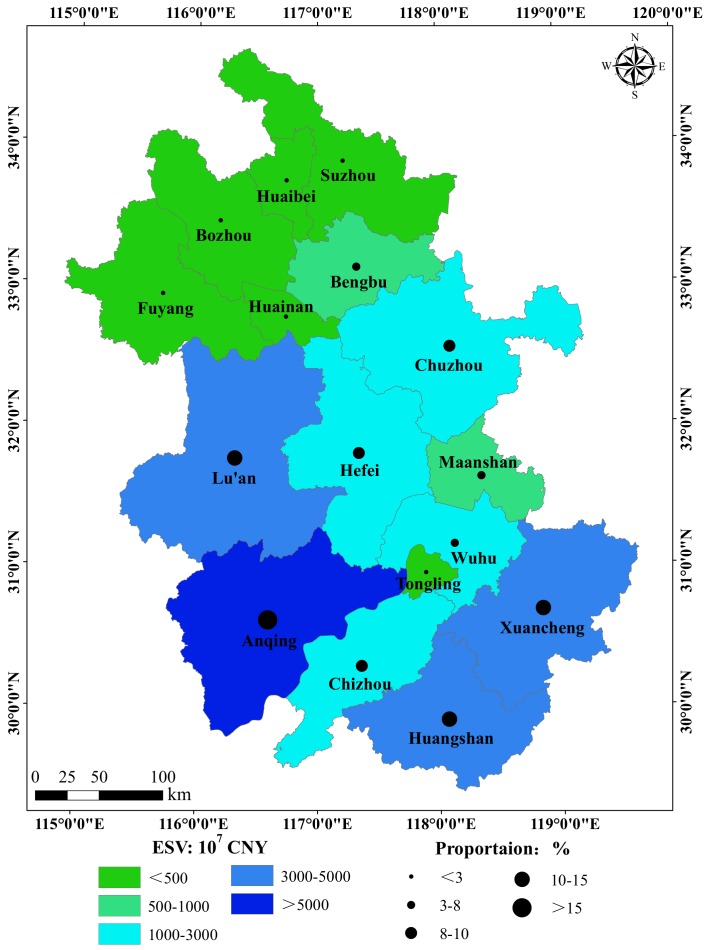
Distribution of total ESVs and their proportions in the 16 cities.

Table 9 shows that the proportions of ESVs in 16 cities in four periods were approximately the same; therefore, we used 2015 as an example to analyze the distribution pattern of the ESV in Anhui province. Anqing has the largest water area and a relatively high proportion of woodlands. In addition, the ESV per unit of water area is much higher than that for other land-use types [44,45]. Therefore, Anqing has the largest ESV in Anhui province, which is consistent with the results we observed in Figure 6. Lu’an, Xuancheng, and Huangshan have the next highest ESVs, with their ESV proportions being 14.59%, 11.57%, and 10.63%, respectively. These three cities were all characterized by large areas of forest land and water area, of which Huangshan had the largest area of forest land area of 7529.94 km^2^. Chaohu Lake is located in Hefei and Chuzhou, and the lower reaches of the Yangtze River flow through Chizhou and Wuhu, which are reasons for their relatively high ESVs. The proportions of farmland in the cities distributed in northern Anhui province were close to 80%, which greatly decreased their total ESV. Built-up land has a significant negative effect on the ESV [2,31], which leads to a relatively low ESV in areas with a large proportion of built-up land. In particular, in Hefei, the built-up land proportion is 17.60%. Although the built-up land proportion in Hefei was relatively large, the water area distributed in this city further increased the total ESV. In general, built-up land, forest land, and water areas are key land-use types that determine the total ESV. The ESV in Anhui province is concentrated in areas with large proportions of water area and forest land, and a low proportion of built-up land.

### 4.2. Effects of Land-Use Change on ESV

In general, land-use intensity has a negative spatial spillover effect on the ESV [46], which indicates that an increase in land-use intensity may lead to the degradation of the ESV of the surrounding areas [41]. Areas with high land-use intensity, such as built-up land and farmland, usually have a low ESV. In contrast, areas with low land-use intensity, such as wet land and water areas, usually have a large ESV [47]. From 2000 to 2015, the land-use intensity of Anhui province continuously increased, which was caused by the continuous expansion of built-up land. However, the ESV continued to decline over the period 2000–2015 and reached its lowest value in 2015. Therefore, we believe that the land-use change caused by urbanization has had a significant impact on the ESV [48]. The urbanization process accelerated rapidly from 2000 to 2015, and the landscape pattern underwent considerable changes. In particular, a large amount of farmland was converted to built-up land; farmland decreased by 4.65% while built-up land increased by 6.86%, resulting in a decrease in the ESV of 692.31 × 10^7^ CNY. The implementation of the project of returning farmland to water areas led to the conversion of a large amount of farmland to water area with an increase in the ESV of 841.48 × 10^7^ CNY. For the entire study area, the total ESV decreased by 331.84 × 10^7^ CNY from 2000 to 2015, and the rate of change was only –1.11%. However, the considerable changes in the ESV for single land-use types should not be ignored. The increment and reduction caused by the expansion of water areas and built-up land areas further balanced the profit and loss of the ESV.

### 4.3. Policy Recommendations

Based on the above findings, we can further propose strategies for ecological planning and management. Changes in the ESV are closely related to land-use change, and the development of urbanization has a negative impact on the ESV [49]. With the rapid economic development in Anhui province, the land-use structure has significantly changed, and the regional ecological balance has been destroyed, which has made this area ecologically fragile [32]. The government should further coordinate socio-economic development and ecological protection, rationally control the expansion of built-up land, and strengthen the protection of ecological land [50]. In the current land-use process, economic benefits inevitably take precedence over ecological benefits [51]. However, the economic losses caused by the neglect of ecological protection may exceed the economic benefits of land use. Therefore, ecological compensation has significant importance in land-use planning and decision-making, as well as in ecosystem services [52]. There are spatial differences in the supply of and demand for ecosystem services, where the demand is mainly distributed in urban areas, and the supply is mainly concentrated in ecological areas. Because the economic development level in ecosystem service supply areas is lower than that in demand areas, decision-makers need to develop effective ecological compensation plans to establish a balance between ecological protection and economic development [12].

### 4.4. Limitations

In this study, we obtained the land-use type maps by interpreting Landsat remote sensing images. Because of our interpretation of the remote sensing images, and the time and space constraints of land-use classification, we only obtained the ESV for a single point in time. Moreover, the resolution of the Landsat data that we used in this study is limited, which affected the accuracy of land-use type classification and the ESV estimation. Therefore, remote sensing images with high resolution should be used in future ESV studies to improve the accuracies of land-use classification and the ESV estimation [53]. The ESV estimation method used in this study was proposed by Costanza et al. [4] and modified by Xie et al. [23], which calculates ESV by multiplying the specific land-use type area and corresponding ESV per unit area and may be affected by many factors, including market price, inflation rate, and government policy [54]. In addition, the ESV we calculated is static in time and space and we did not consider the heterogeneity of equivalent biomes [55,56]. These limitations should be addressed in subsequent studies. Nevertheless, this study focused on the spatiotemporal variation in the ESV and its response to land-use change; therefore, the results obtained are considered reasonable and reliable.

## 5. Conclusions

In this study, we characterized the land-use change in Anhui Province from 2000 to 2015 using remote sensing image data. In addition, we discussed the impact of land-use change on the ESV. The key findings and main conclusions are summarized as follows:

- Paddy field represented the largest proportion of land-use types in Anhui province, followed by unirrigated field and forest land. The acceleration of urbanization is the main driving force for the transformation of land-use types, and the main trend of land-use change is a significant increase in built-up land (3768.07 km^2^) and a significant decrease in farmland (−3751.16 km^2^). Farmland is the most important transfer source of built-up land.
- From 2000 to 2015, the total ESV of Anhui province decreased by 331.84 × 10^7^ CNY. Among the 11 ecosystem service types, hydrological regulation made the largest ESV contribution, accounting for approximately 50% of the total ESV. Water supply was the only ecological service type that had a negative effect on ESV, which is closely related to the expansion of built-up land and a large proportion of paddy field. Among the 16 cities, Anqing had the largest proportion of water area, which led to the largest ESV in this city. However, the ESVs of cities with high urbanization rates, such as Hefei, Maanshan, and Wuhu, have been greatly affected.
- The acceleration of urbanization has promoted the expansion of built-up land, resulting in a decrease in the ESV of 765.10 × 10^7^ CNY. The expansion of water areas led to an increase in ESV of 1175.37 × 10^7^ CNY. Although the land-use change caused a significant change in ESV, the negative effect of built-up land on ESV covered this feature.

In general, urbanization-induced land-use change has an important impact on ESV. The evaluation of land-use change and ESV is extremely helpful for the coordination of socio-economic development and ecological protection. The results of this study may help decision-makers to develop land-use planning and environmental protection policies in Anhui province.

## Figures and Tables

**Table 1:** Landsat image data used in this study.

Year	Sensor	Acquisition Date (Path/Row)
2000	TM	2000-10-10 (120/37), 2000-10-10 (120/38), 2000-10-10 (120/39), 2000-10-10 (120/40), 2000-11-02 (121/36), 2000-11-02 (121/37), 2000-11-02 (121/38), 2000-11-02 (121/39), 2000-09-22 (122/36), 2000-09-22 (122/37), 2000-10-08 (122/38), 2000-10-08 (122/39), 2000-10-15 (123/36), 2000-10-15 (123/37)
2005	TM	2005-10-24 (120/37), 2005-10-24 (120/38), 2005-10-24 (120/39), 2005-10-24 (120/40), 2005-10-31 (121/36), 2005-10-31 (121/37), 2005-10-31 (121/38), 2005-10-31 (121/39), 2005-11-07 (122/36), 2005-11-07 (122/37), 2005-11-07 (122/38), 2005-11-07 (122/39), 2005-10-29 (123/36), 2005-10-29 (123/37),
2010	ETM+	2010-10-30 (120/37), 2010-10-30 (120/38), 2010-10-30 (120/39), 2010-10-30 (120/40), 2010-10-05 (121/36), 2010-10-05 (121/37), 2010-10-05 (121/38), 2010-10-05 (121/39), 2010-10-28 (122/36), 2010-10-28 (122/37), 2010-10-28 (122/38), 2010-10-28 (122/39), 2010-11-04 (123/36), 2010-11-04 (123/37)
2015	OLI	2015-10-20 (120/37), 2015-10-20 (120/38), 2015-10-20 (120/39), 2015-10-20 (120/40), 2015-10-11 (121/36), 2015-10-11 (121/37), 2015-10-11 (121/38), 2015-10-11 (121/39), 2015-10-02 (122/36), 2015-10-02 (122/37), 2015-10-02 (122/38), 2015-10-02 (122/39), 2015-10-09 (123/36), 2015-10-09 (123/37)

TM: Thematic Mapper; ETM+: Enhanced Thematic Mapper Plus; OLI: Operational Land Imager.

**Table 2:** The equivalent factor of ecosystem service value (ESV) per unit area in Anhui province.

Ecosystem Service	Type	Paddy Field	Unirrigated Field	Forest Land	Grass Land	Water Area	Wet Land	Built-Up Land	Unused Land
Provisioning services	Food production (FP)	1.36	0.85	0.29	0.38	0.80	0.51	0.01	0.00
	Raw material production (RMP)	0.09	0.40	0.66	0.56	0.23	0.50	0.00	0.00
	Water supply (WS)	−2.63	0.02	0.34	0.31	8.29	2.59	−7.51	0.00
Regulating services	Gas regulation (GR)	1.11	0.67	2.17	1.97	0.77	1.90	−2.42	0.02
	Climate regulation (CR)	0.57	0.36	6.50	5.21	2.29	3.60	0.00	0.00
	Hydrological regulation (HR)	2.72	0.27	4.74	3.82	102.24	24.23	0.00	0.03
	Environmental purification (EP)	0.17	0.10	1.93	1.72	5.55	3.60	−2.46	0.10
Supporting services	Soil formation and retention (SR)	0.01	1.03	2.65	2.40	0.93	2.31	0.02	0.02
	Maintain nutrient cycling (MNC)	0.19	0.12	0.20	0.18	0.07	0.18	0.00	0.00
	Biodiversity protection (BP)	0.21	0.13	2.41	2.18	2.55	7.87	0.34	0.02
Cultural services	Recreation and culture (RC)	0.09	0.06	1.06	0.96	1.89	4.73	0.01	0.01
	**Total**	3.89	4.01	22.95	19.69	125.61	52.02	−12.01	0.20

**Table 3:** ESVs per unit area in Anhui province (Yuan/hm^2^/year).

Ecosystem Service	Type	Paddy Field	Unirrigated Field	Forest Land	Grass Land	Water Area	Wet Land	Built-Up Land	Unused Land
Provisioning services	FP	2139.89	1337.43	456.30	597.91	1,258.76	802.46	15.73	0.00
	RMP	141.61	629.38	1038.48	881.13	361.89	786.73	0.00	0.00
	WS	−4138.17	31.47	534.97	487.77	13,043.90	4075.24	−11,816.61	0.00
Regulating services	GR	1746.53	1054.21	3414.39	3099.70	1211.56	2989.56	−3807.75	31.47
	CR	896.87	566.44	10,227.43	8197.67	3603.20	5664.42	0.00	0.00
	HR	4279.78	424.83	7458.15	6010.58	160,869.53	38,124.69	0.00	47.20
	EP	267.49	157.35	3036.76	2706.33	8732.65	5664.42	−3870.69	157.35
Supporting services	SR	15.73	1620.65	4169.64	3776.28	1463.31	3634.67	31.47	31.47
	MNC	298.96	188.81	314.69	283.22	110.14	283.22	0.00	0.00
	BP	330.42	204.55	3792.01	3430.12	4012.30	12,383.05	534.97	31.47
Cultural services	RC	141.61	94.41	1667.86	1510.51	2973.82	7442.42	15.73	15.73
	Total	6120.72	6309.53	36,110.68	30,981.23	197,641.05	81,850.87	18,897.13	314.69

**Table 4:** Dynamic degrees of change in land-use type from 2000 to 2015 (%).

Land-Use Type	Single Land-Use Dynamic Degree			
	2000–2005	2005–2010	2010–2015	2000–2015
Paddy field	−0.11	−0.42	−0.55	−3.54
Unirrigated field	−0.07	−0.26	−0.45	−2.57
Forest land	0.00	−0.05	−0.11	−0.51
Grass land	−0.01	−0.07	−0.14	−0.75
Water area	0.14	0.16	1.06	4.58
Wet land	−0.06	0.06	−2.13	−7.08
Built-up land	0.55	2.47	2.94	21.63
Unused land	0.10	1.59	95.97	352.75
Comprehensive land-use dynamic degree	0.06	0.17	0.26	0.14

**Table 5:** Land-use transition matrix of Anhui province from 2000 to 2015 (km^2^).

2000	2015								Total
	Paddy Field	Unirrigated Field	Forest Land	Grass Land	Water Area	Wet Land	Built-Up Land	Unused Land	
Paddy field	41,012.80	42.57	146.01	36.64	310.48	13.50	2286.79	12.71	43,861.49
Unirrigated field	87.83	35,256.18	24.14	9.99	115.28	37.63	1376.80	2.67	36,910.52
Forest land	152.43	20.72	31,786.97	56.25	20.82	1.91	241.30	6.11	32,286.51
Grass land	22.61	4.31	71.33	8170.79	13.51	1.21	88.79	2.72	8375.27
Water area	73.18	22.30	4.55	2.73	5914.88	28.47	45.31	0.33	6091.74
Wet land	62.62	9.37	0.83	0.61	118.87	898.54	9.52	0.00	1100.36
Built-up land	117.69	131.65	4.39	3.29	15.74	2.04	11,254.11	1.11	11,530.02
Unused land	0.01	0.00	0.06	0.03	0.00	0.00	0.24	4.42	4.77
Total	41,529.16	35,487.11	32,038.27	8280.32	6509.58	983.30	15,302.86	30.06	140,160.68

**Table 6:** The land-use intensity of Anhui province from 2000 to 2015.

Year	2000	2005	2010	2015
Intensity	274.13	274.33	275.43	276.81

**Table 7:** ESVs of Anhui province in 2000, 2005, 2010, and 2015 (10^7^ CNY/year).

Land-Use Type	2000	2005	2010	2015	2000–2005 (%)	2005–2010 (%)	2010–2015 (%)	2000–2015 (%)
Paddy field	2681.67	2667.09	2610.45	2539.09	−0.54	−2.12	−2.73	−5.32
Unirrigated field	2326.24	2318.40	2287.89	2236.53	−0.34	−1.32	−2.24	−3.86
Forest land	11,659.07	11,661.60	11,630.81	11,569.64	0.02	−0.26	−0.53	−0.77
Grass land	2595.66	2593.94	2584.75	2566.42	−0.07	−0.35	−0.71	−1.13
Water area	12,046.89	12,131.94	12,226.04	12,873.71	0.71	0.78	5.30	6.86
Wet land	900.98	898.20	901.04	805.25	−0.31	0.32	−10.63	−10.63
Built-up land	−2194.94	−2255.47	−2534.19	−2907.00	2.76	12.36	14.71	32.44
Unused land	0.02	0.02	0.02	0.09	0.49	7.97	479.84	529.13
**Total**	30,015.58	30,015.71	29,706.83	29,683.74	0.00	−1.03	−0.08	−1.11

**Table 8:** Profit and loss matrix for the ESV in Anhui province from 2000 to 2015 (10^7^ CNY).

2000	2015								Total
	Paddy Field	Unirrigated Field	Forest Land	Grass Land	Water Area	Wet Land	Built-Up Land	Unused Land	
Paddy field	——	2.69	52.72	11.35	613.64	11.05	−432.14	0.04	259.35
Unirrigated field	5.38	——	8.72	3.09	227.84	30.80	−260.18	0.01	15.66
Forest land	9.33	1.31	——	17.43	41.14	1.56	−45.60	0.02	25.19
Grass land	1.38	0.27	25.76	——	26.70	0.99	−16.78	0.01	38.34
Water area	4.48	1.41	1.64	0.85	——	23.30	−8.56	0.00	23.12
Wet land	3.83	0.59	0.30	0.19	234.93	——	−1.80	0.00	238.04
Built-up land	7.20	8.31	1.58	1.02	31.11	1.67	——	0.00	50.89
Unused land	0.00	0.00	0.02	0.01	0.01	0.00	−0.05	——	0.00
Total	31.61	14.57	90.75	33.93	1175.37	69.38	−765.10	0.08	650.59

**Table 9:** ESVs and their proportions in the 16 cities (10^7^ CNY; %).

District	2000		2005		2010		2015	
	ESV	Proportion	ESV	Proportion	ESV	Proportion	ESV	Proportion
Anqing	5648.79	18.87	5642.09	18.85	5598.65	18.90	5645.68	19.07
Bengbu	952.71	3.18	951.67	3.18	943.53	3.19	923.09	3.12
Bozhou	308.69	1.03	294.85	0.99	277.58	0.94	257.94	0.87
Chizhou	2795.93	9.34	2800.23	9.36	2770.38	9.35	2752.98	9.30
Chuzhou	2534.28	8.47	2554.99	8.54	2542.84	8.58	2609.48	8.81
Fuyang	389.00	1.30	381.98	1.28	361.55	1.22	406.79	1.37
Hefei	2618.41	8.75	2600.39	8.69	2538.17	8.57	2555.14	8.63
Huaibei	179.03	0.60	173.54	0.58	164.60	0.56	145.53	0.49
Huainan	385.06	1.29	387.63	1.30	389.06	1.31	388.05	1.31
Huangshan	3165.26	10.57	3166.75	10.58	3150.60	10.64	3148.33	10.63
Lu’an	4290.82	14.33	4271.34	14.27	4263.01	14.39	4318.79	14.59
Maanshan	1016.03	3.39	1051.04	3.51	1060.58	3.58	991.49	3.35
Tongling	370.59	1.24	372.64	1.25	353.08	1.19	336.92	1.14
Wuhu	1316.92	4.40	1326.17	4.43	1292.88	4.36	1267.47	4.28
Suzhou	472.28	1.58	467.10	1.56	461.01	1.56	431.42	1.46
Xuancheng	3491.18	11.66	3487.90	11.65	3455.18	11.66	3424.83	11.57
